# Effect of ketorolac tromethamine combined with dezocine prior administration on hemodynamics and postoperative analgesia in patients undergoing laparoscopic hernia repair

**DOI:** 10.1097/MD.0000000000029320

**Published:** 2022-05-27

**Authors:** Yu Wu, Zenghua Cai, Yanli Li, Yuling Kang, Bohan Fu, Jinbao Wang

**Affiliations:** Department of Anesthesiology, No. 980 Hospital of Joint Logistic Support Force (Bethune International Peace Hospital), Shijiazhuang, China.

**Keywords:** hernia repair, ketorolac tromethamine, laparoscopy, preventive analgesia

## Abstract

**Objective:**

To observe the effect of Ketorolac tromethamine combined with dezocine prior administration on hemodynamics and postoperative sedation in patients undergoing laparoscopic hernia repair.

**Methods:**

100 male patients aged 60 to 80 years old, a line to elective laparoscopic inguinal hernia repair, were randomly divided into four groups: control group (Group A) and dezocine group (Group B), ketorolac tromethamine group (Group C), ketorolac tromethamine combined with dezocine group (Group D). Patients were administrated with 0.1 mg/kg dezocine in Group B, 0.5 mg/kg ketorolac in Group C, 0.1 mg/kg dezocine, and 0.5 mg/kg ketorolac in Group D, and with an equal dose of normal saline in group A. The heart rate (HR) and mean arterial pressure (MAP) of patients in 4 groups were recorded at each time point as follows, T0 (enter the operating room), T1 (before skin resection), 10 min after pneumoperitoneum (T2), mesh placement (T3), and laryngeal mask extraction (T4). Operation time, awakening time (time from drug withdrawal to consciousness recovery), the dosage of propofol, sufentanil, remifentanil, and intraoperative vasoactive drug dosage were recorded to compare. Visual analog scale score and sedation Ramsay score were evaluated 1, 6, 12, and 24 hours after extubation.

**Results:**

There was no significant difference in operation time, anesthesia recovery time, sufentanil dosage, and vasoactive drugs among all groups. The amount of propofol in Group B and D was less than that in Group A and C (*P* < .05), and there was no difference between Group B and D, A and C (*P* > .05). The amount of remifentanil in Group B, C, and D was less than that in Group A (*P* < .05), and Group D was less than B and C (*P* < .05). After extubation, HR and MAP were significantly higher than before (*P* < .05). Compared with T0, HR and MAP increased in each group at T4, but MAP and HR in Group D increased the least (*P* < .05). There were significant differences between Group B, C, D, and A, MAP and HR fluctuated little during extubation (*P* < .05), but there was a significant difference between Group D and B, C (*P* < .05). Visual analog scale scores of Group B, C, and D were lower than those of A at 1, 6, and 12 hours after surgery (*P* < .05), and there was a significant difference between Group D, and B, C (*P* < .05). Ramsay scores in Group B and D were higher than those in A and C at 1 and 6 hours after the operation (*P* < .05). There was no difference in the incidence of adverse reactions among groups.

**Conclusion:**

The prophylactic use of ketorolac tromethamine and dezocine before laparoscopic inguinal hernia repair can reduce hemodynamic disorder during anesthesia recovery, increase postoperative sedative and analgesic effects.

## 1 Introduction

Laparoscopic inguinal hernia repair is a popular tension-free repair operation in recent years, which mainly includes two methods: transabdominal preperitoneal transplantation and extraperitoneal transplantation.^[1]^ Laparoscopic herniorrhaphy has a small, aesthetically invasive incision and enables the exploration of contralateral hernia, the detection of occulted and femoral hernias, with postoperative recovery faster, the recurrence rate lower, pain and discomfort rate less compared to open herniorrhaphy.^[2]^ It reduces a 1.28 days hospital stay, and $2400 hospitalization costs compare to traditional hernia repair.^[3]^ Laparoscopic inguinal hernia repair requires general anesthesia at an appropriate depth to make the patients tolerate laparoscopy and pneumoperitoneum without delay in recovery. In recent years, with the enhanced recovery after surgery and concept of preventive analgesia, especially multi-mode preventive analgesia, the body's nervous and endocrine system were preventively interposed before the harmful stimulation subjected to the body. Reduce the adverse effects of harmful stimuli on the body,^[4]^ and bring a new vision to clinical anesthesia.

Ketorolac tromethamine is a non-steroidal anti-inflammatory drug that can inhibit the biosynthesis of prostaglandins and has analgesic effects.^[5]^ Dezocine, a partial μ receptor agonist and κ receptor antagonist, is widely used in postoperative analgesia and pain treatment of abdominal organs.^[6]^ To optimize intraoperative anesthesia in patients with intraoperative and postoperative analgesia drug use, promote accelerated rehabilitation patients. To the best of our knowledge, this is the first attempt to observe the anesthetic effect, perioperative sedation, and analgesia of patients undergoing laparoscopic inguinal hernia repair by preoperative intravenous administration of ketorolac tromethamine combined with dezocine.

## 2 Methods

### 2.1 Study design

**Figure 1:**
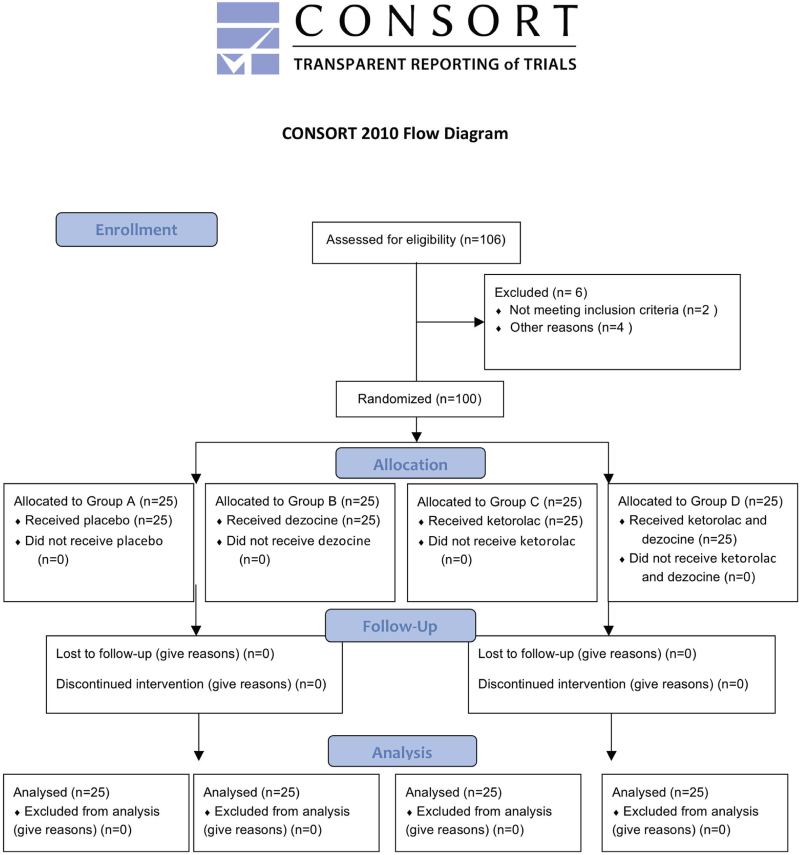
The CONSORT 2010 flow diagram describing patient progress through the study.

This study was approved by the No.980 Hospital Ethics Committee (Ethics No. 2021-KY-127), and assume that the ratio of the two groups is 1:1, using a two-sided test with a significance level (α) of 0.05 and a power (1-β) of 0.80, the standard deviation of the sample was 0.15 and the required sample size was 25 in each group.^[7,8]^ Therefore, 106 patients undergoing laparoscopic inguinal hernia repair under elective general anesthesia from August to December in 2021 were selected and obtained patient's consent and signed informed consent, 6 patients were excluded (Fig. 1). Patients were randomly divided into 4 groups (n = 25) using sequentially coded, opaque, sealed envelopes: control group (Group A), dezocine group (Group B), ketorolac group (Group C), and ketorolac combined with dezocine group (Group D). The inclusion criteria for this study: were American Society of Anesthesiologists grade I-II, male, age 60 to 80 years old, body mass index between 20 and 30 kg/m^2^. Exclusion criteria: patients with cognitive dysfunction, severe cardiac disease (severe arrhythmia, double-bundle branch block), severe sinoatrial node dysfunction, heart failure, severe renal diseases, long-term use of sedatives, antidepressants, and opioids, gabapentinoids and other chronic pain medications, history of gastrointestinal ulcer, ketorolac or dezocine allergy.

There were no statistically significant differences in age, body mass index, American Society of Anesthesiologists grade, and co-history (mild heart disease and hypertension) among the 4 groups (*P* > .05), as shown in Table 1.

**Table 1:** general demographic characteristic of patients.

group	A	B	C	D
Age (year)	67.56 ± 4.21	66.64 ± 3.79	65.76 ± 3.72	67.04 ± 3.60
BMI (kg/m^2^)	24.32 ± 0.42	24.25 ± 0.53	24.00 ± 0.48	24.31 ± 0.32
ASA (case)(I/II)	9/16	10/15	12/13	11/14
mild heart disease (Y/N)	7/18	8/17	7/18	6/19
Hypertension (Y/N)	7/18	9/16	6/19	8/17

ASA = American Society of Anesthesiologists, BMI = body mass index.

### 2.2 Anesthesia procedure

The patient was given atropine 0.5 mg and diazepam 10 mg intramuscular injection 30 minute before surgery. Entering the operating room, the patient was routinely given lactate ringer's fluid through the peripheral vein. Electrocardiogram, Pulse oximetry (SpO_2_), heart rate (HR), non-invasive blood pressure, respiratory rate, pulmonary end-tidal CO_2_ (PetCO_2_), and bispectral index were monitored. Ketorolac (Chengdu Beite Pharmaceutical Co., LTD., Batch No. 210302), dezocine (Yangzijiang Pharmaceutical Group Co., LTD., Batch No. 21031131), ketorolac group (Group C) and ketorolac combined with dezocine group (Group D) were intravenously given 0.5 mg kg-1 ketorolac before anesthesia induction. The other two groups were given the same amount of normal saline. Anesthesia induction under mask ventilation: midazolam 0.05 mg kg^−1^, cisatracurium 0.15 mg  kg^−1^, etomidate 0.2 to 0.4 mg  kg^−1^, sufentanil 0.2 to 0.3ug  kg^−1^. Mechanical ventilation was performed by placing a single-cavity laryngeal mask after the patient's jaw relaxed, using volumetric control ventilation and setting respiratory parameters: tidal volume 6 to 8 mL kg^−1^, respiratory rate 12 to 16 times min^−1^. During the operation, respiratory parameters were adjusted according to the pneumoperitoneum, and PetCO_2_ was maintained between 35 to 45 mm Hg (1mm Hg = 0.133kPa). After induction of anesthesia, patients in the dezocine group (Group B) and ketorolac combined with the dezocine group (Group D) were given intravenous dezocine 0.1 mg kg-1, and the other two groups were given the same amount of normal saline. Anesthesia maintenance: all patients were given total intravenous anesthesia: propofol 100 to 150 μg/(kg min)^−1^ and remifentanil 0.1 to 0.2 μg/(kg min)^−1^ to maintain the bispectral index value between 40 to 60, and add 0.05 mg kg^−1^ cisatracurium every 30 minutes intraoperatively for muscular flaccidity. Corresponding vasoactive drugs (ephedrine, atropine) were given when the patients hemodynamically were unstable. Propofol and remifentanil were stopped immediately after surgery and tropisetron 5 mg was given intravenously to prevent postoperative nausea and vomiting.^[9]^ After the surgery, the patient was given neostigmine 1 mg and atropine 0.5 mg for muscle relaxation antagonism until spontaneous respiration recovered. After waking up, the laryngeal mask was removed (indications: self-opening eyes, recovery of swallowing reflex, RR > 12 times /min, tidal volume > 5 mL kg^−1^, SpO2 > 95% after 5 min of self-breathing without oxygen), and sent to the post-anesthesia recovery room.

### 2.3 Outcome measures

The HR, mean arterial pressure (MAP), operation time, time to wake up (time from drug withdrawal to eye-opening), the total volume of propofol, sufentanil, and remifentanil were recorded during surgery. Visual analog scale (VAS) and Ramsay scores at 1, 6, 12, and 24 h after extubation were also recorded. VAS scoring standard^[10]^:0, painless; 1–3, mild pain, a patient can tolerate; 4–6, patients can tolerate pain and sleep disturbance; 7–10, patients have progressively intense pain that is unbearable. Ramsay scoring standard^[11]^: 1, anxiety, agitation; 2, accurate orientation, quiet to cooperate with the operation; 3. responds only to commands. 4, quick response to strong sound stimulation and tapping eyebrow; 5, insensitive to strong sound stimulation and tapping eyebrow; 6, no response to strong sound stimulation and tapping eyebrow.

### 2.4 Statistical analysis

Prism 8.0 statistical software was used, and measurement data were expressed as mean ± standard deviation. Analysis of variance was used for inter-group comparison and analysis of variance for intra-group comparison. Statistical data were expressed as percentage (%) and χ^2^ test was used for comparison between groups. *P* < .05 was considered statistically significant.

## 3 Results

### 3.1 Variation of MAP, HR, and use of vasoactive drugs at each time point

There were no significant differences in total operation time (standard deviation [SD], 59.48[3.60] vs 61.76[3.09] vs 61.60[3.61] vs 59.24[3.35]), recovery time (SD, 11.58[1.95] vs 11.13[1.70] vs 11.17[2.10] vs 10.83[2.26]) and the use of vasoactive drugs (ephedrine and atropine) among all groups (*P* > .05). The dosage of propofol among all groups showed that Group B (SD, 284.8[12.62]) was less than Group A (SD, 299.2[23.39]) and C (SD, 298.8[19.65]), and Group D (SD, 283.6[20.23]) was less than group C, with statistically significant differences (*P* < .05), as shown in Table 2. The HR and MAP decreased with anesthesia induction but increased with CO_2_ pneumoperitoneum. When patients adapted to CO_2_ pneumoperitoneum implantation, the HR and MAP decreased to a certain extent. After extubation, the HR and MAP in each group were significantly higher than before (*P* < .05). Compared within the group at the T0 time point, the MAP and HR in groups B, C, and A were significantly increased at T4 (*P* < .05), while no significant changes were observed in group D (*P* > .05). At the T4 time point, MAP and HR of groups B and C were lower than those of group A (*P* < .05), but still higher than those of group D (*P* < .05), as shown in Table 3.

**Table 2:** Surgical baseline clinical characteristics.

Group	A	B	C	D
Operation time(min)	59.48 ± 3.60	61.76 ± 3.09	61.60 ± 3.61	59.24 ± 3.35
Propofol (mg)	299.2 ± 23.39^d^	284.8 ± 12.62^a^	298.8 ± 19.65^d^	283.6 ± 20.23^a^
Sufentanil(ug)	18.04 ± 1.27	17.88 ± 1.17	17.32 ± 1.15	17.84 ± 1.11
Remifentanil(ug)	799.2 ± 88.31^d^	743.6 ± 57.29^a^^,^^d^	736.4 ± 69.57^a^^,^^d^	682.0 ± 65.51^a^
Recovery time(min)	10.83 ± 2.26	11.13 ± 1.70	11.17 ± 2.10	11.58 ± 1.95
Ephedrine (Y/N)	2/23	3/22	2/23	4/21
Atropine(Y/N)	4/21	5/20	3/22	4//21

a Compared to group A, *P* < .05.

d Compared to group D, *P* < .05. N = no, Y = yes.

**Table 3:** MAP and HR variation in patients during operation.

Group	A	B	C	D
HR (Beats per min)				
T0	72.36 ± 5.02	74.04 ± 5.37	73.44 ± 5.03	73.96 ± 5.43
T1	68.16 ± 4.71	69.44 ± 4.90	69.00 ± 4.62	69.44 ± 4.90
T2	83.16 ± 5.78	83.56 ± 5.99	82.44 ± 6.23	80.20 ± 5.44
T3	77.60 ± 5.36	78.04 ± 5.53	77.04 ± 5.63	74.96 ± 5.11
T4	96.48 ± 6.43^∗^^,^^d^	92.32 ± 4.91^∗^^,^^a^^,^^d^	92.16 ± 5.91^∗^^,^^a^^,^^d^	87.24 ± 4.65^∗^^,^^a^
MAP(mm Hg)				
T0	75.88 ± 5.44	74.76 ± 4.70	74.36 ± 3.58	77.52 ± 4.06
T1	73.28 ± 5.05	72.16 ± 4.32	71.84 ± 3.21	74.76 ± 3.79
T2	84.80 ± 6.09	83.60 ± 5.27	83.08 ± 4.03	86.68 ± 4.45
T3	83.88 ± 6.10	82.64 ± 5.32	82.28 ± 3.89	85.76 ± 4.43
T4	86.36 ± 5.30^∗^^,^^d^	82.68 ± 5.36^∗^^,^^a^^,^^d^	82.16 ± 4.09^∗^^,^^a^^,^^d^	77.52 ± 4.06^a^

∗ Compared with T0, *P*< .05.

a Compared with A, *P* < .05.

d Compared with D, *P* < .05.

### 3.2 Sedation and analgesia score

At the end of the surgery, the pain score of all patients gradually increased, which may be related to the complete metabolism of troublesome analgesic drugs, but the pain stimulating factors in the body have not been completely eliminated. At the same time, compared with Group A, VAS scores at 1, 6, and 12 hours after surgery were lower in Group B and Group C (*P* < .05), but higher than Group D (*P* < .05), and there was no statistical significance between Group B and Group C (*P* > .05). At the same time, the Ramsay score of all patients peaked at 1 hour after surgery and gradually decreased with the passage of time. At the time point 1 and 6 hours after surgery, the Ramsay score of Group D increased significantly compared with group A (*P* < .05), as shown in Table 4.

**Table 4:** VAS and Ramsay score at each postoperative time point.

group	A	B	C	D
VAS scores				
1h	4.72 ± 1.26^b^^,^^c^^,^^d^	3.52 ± 1.05^a^^,^^d^	3.48 ± 1.12^a^^,^^d^	2.64 ± 0.95^a^^,^^b^^,^^c^
6h	4.80 ± 1.15^b^^,^^c^^,^^d^	3.76 ± 1.05^a^^,^^d^	3.84 ± 1.28^a^^,^^d^	2.80 ± 1.00^a^^,^^b^^,^^c^
12h	4.84 ± 0.97^b^^,^^c^^,^^d^	4.00 ± 0.65^a^^,^^d^	3.88 ± 1.13^a^	3.24 ± 1.05^a^^,^^b^
24h	5.32 ± 1.46	4.84 ± 0.94	4.68 ± 1.49	3.84 ± 1.55^a^
Ramsay scores				
1h	1.76 ± 0.66	2.28 ± 0.74	1.84 ± 0.94	2.48 ± 0.92^a^^,^^c^
6h	1.64 ± 0.95	2.24 ± 0.88	1.80 ± 0.96	2.44 ± 0.87^a^
12h	1.56 ± 0.92	2.20 ± 0.91	1.88 ± 1.01	2.36 ± 1.04^a^
24h	1.48 ± 0.87	1.68 ± 0.90	1.64 ± 0.95	1.72 ± 0.98

a Compared with A, *P* < .05.

b Compared with B, *P* < .05.

c Compared with C, *P* < .05.

d Compared with D, *P* < .05.

### 3.3 Adverse events

Complications of laparoscopic inguinal hernia repair include pain in the operative site and groin area, postoperative respiratory depression, postoperative nausea and vomiting after anesthesia, postoperative urinary retention, bladder injury, and scrotal hematoma, among which the scrotal or groin hematoma is the most common, with an incidence of 0.5% to 78%.^[12]^ This study found that the incidence of respiratory depression and nausea and vomiting in patients after laparoscopic inguinal hernia repair was about 8%, and there was no statistical significance among all groups (*P* > .05), as shown in Table 5. In this study, a total of 1 case of surgical site hematoma occurred, and the patient was in group B.

**Table 5:** Incidence of postoperative adverse reactions (cases, %).

group	A	B	C	D
Respiratory depression	2 (8)	3 (12)	3 (12)	4 (16)
PONV	2 (8)	3 (16)	2 (8)	4 (16)
Surgical site hematoma	0 (0)	1 (4)	0 (0)	0 (0)

PONV = postoperative nausea and vomiting.

## 4 Discussion

In the present study, patients undergoing hernia repair under general anesthesia were selected for total intravenous anesthesia, and the effects of ketorolac tromethamine or dezocine alone or in combination on intraoperative hemodynamics and postoperative analgesia and sedation were considered. Hernia repair can stimulate the body to produce 5-hydroxytryptamine,^[13]^ bradykinin,^[14]^ histamine,^[15]^ and other bioactive substances, as well as the accumulation of prostaglandin^[16]^ and oxidative stress,^[17]^ which will greatly enhance the pain caused by these substances. Although the incidence of pain in laparoscopic inguinal hernia repair is lower than that of traditional open surgery, the incidence of postoperative chronic pain is 12.7%, of which 83.3% of patients’ pain is located in the groin area.^[18]^ The occurrence of pain is related to mesh size, whether the mesh is mechanically fixed, bleeding and infection at the surgical site, etc.^[19]^. In recent years, anesthesiologists have gradually noticed that preventive analgesia can bring good benefits in many clinical scenarios.^[20]^

Remifentanil, as an ultra-short-acting μ receptor agonist, is widely used in total intravenous anesthesia because it takes only 3 to 10 minutes to achieve an effective concentration of action and has a very short half-life.^[21]^ Because of its characteristics, if remifentanil is used alone during the perioperative period, especially at the end of the surgery, it is easy to cause agitation during anesthesia recovery and perioperative acute pain,^[22]^ increasing agitation and hemodynamic disorder during anesthesia recovery. At the same time, it will also lead to the occurrence of postoperative pain and rebound pain.^[23]^ There have been reports of prior administration of ketorolac tromethamine and dezocine, and the preoperative preventive intravenous administration of dezocine can effectively prevent and treat hyperalgesia and has a certain analgesic effect, which lasts for 6 to 12 hours.^[24]^ Ketorolac tromethamine can reduce prostaglandin synthesis in patients and relieve pain caused by related pain mediators.^[25]^ The operative time of laparoscopic inguinal hernia repair is not long, and the half-life and pharmacodynamic time of ketorolamine and dezocine can completely cover the entire surgical process, theoretically preventing the occurrence of hyperalgesia related to hernia repair.^[26]^ The dynamic changes of intraoperative blood pressure and heart rate can directly reflect the changes in the hemodynamics of patients, especially at a specific time point of operation. VAS score^[27]^ and Ramsay sedation score^[28]^ are widely used to evaluate perioperative analgesia and sedation levels of patients. Therefore, in this study, preoperative intravenous administration of ketorolac tromethamine and dezocine was selected to reduce surgical stress and reduce the occurrence of hyperalgesia after remifentanil, improve the quality of patients’ recovery and reduce the occurrence of agitation and hemodynamic disorders during patients’ recovery, and the above observation indicators were selected. According to literature reports,^[29]^ the prophylactic use of ketorolac tromethamine in laparoscopic inguinal hernia repair is safe and effective, and dezocine local anesthesia can relieve intraoperative and postoperative pain, improve postoperative comfort,^[30]^ and relieve postoperative agitation in preschool children.^[31]^ However, there have been no reports about the combined effect of ketorolac and dezocine on laparoscopic hernia repair.

The results of this study showed that the analgesic effects of ketorolac tromethamine and dezocine were similar, and both had synergistic analgesic and sedative effects. The use of dezocine, ketorolac tromethamine, and their combination had little effect on the hemodynamics of the patients, and there was no statistically significant difference between the groups. However, the combined use of ketorolac tromethamine and dezocine can reduce the disturbance of mean arterial pressure and heart rate in patients undergoing laparoscopic inguinal hernia repair during anesthesia recovery, and ketorolac tromethamine and dezocine also have a good synergistic effect on maintaining hemodynamic stability during anesthesia recovery. We found that the VAS of patients undergoing laparoscopic inguinal hernia repair was about 5 points at 1 hour after surgery, which also verified the previous report,^[32]^ and the pain level could continue to 24 hours after surgery. Although follow-up found between groups there was no statistically significant difference related complications in patients with anesthesia, but we still need to pay attention to the laparoscopic assisted hernia repair surgery postoperative respiratory depression and the risk of nausea and vomiting is still as high as 10%, but our study is the relatively low incidence of postoperative nausea and vomiting male patients, for female patients,^[33,34]^ We estimate the incidence of postoperative nausea and vomiting to be higher than in our study. Since ketorolac tromethamine is a non-steroidal anti-inflammatory drug, it may cause the risk of coagulation disorder in patients,^[35,36]^ so we strengthened the follow-up awareness of postoperative hematoma at the surgical site of patients. Our results suggest that a single dose of 30 mg of ketorolac tromethamine does not increase the risk of bleeding.

The anesthetic goals of laparoscopic hernia repair surgery include ensuring hemodynamic and respiratory stability, adequate muscle relaxation, management of pneumoperitoneum, postoperative analgesia and early recovery, and minimizing side effects.^[37]^ For laparoscopic inguinal hernia surgery, the establishment of pneumoperitoneum by injecting CO_2_ into the abdominal cavity can also cause pathophysiological changes in the cardiovascular system. The main causes of these changes are related to high pneumoperitoneum pressure, absorption of CO_2_, which leads to hypercapnia (followed by acidosis), and exacerbation of elevated intra-abdominal pressure. Other factors that exacerbate the pathophysiological changes in the cardiovascular system include the patient's position during surgery, surgical conditions, and the patient's fluid volume.^[38]^ During laparoscopic hernia surgery under general anesthesia, the use of individualized controlled ventilation usually prevents hypercapnia and allows for the expulsion of carbon dioxide, although in some cases patients develop severe hypercapnia due to prolonged surgery, and extreme Trendelenburg positions are associated with impaired cardiopulmonary function.^[39]^ Increased intra-abdominal pressure during pneumoperitoneum causes a variety of cardiovascular changes, depending on how much intra-abdominal pressure is achieved. When pressure exceeds 15 mm Hg, an increase in heart rate (mean arterial pressure), systemic and pulmonary vascular resistance, and a decrease in cardiac output are usually noted.^[40]^ At lower inflation pressures (8–12 mm Hg), these hemodynamic changes are lighter. How patients respond to these changes depends on their age, their current clinical condition, and comorbidities.^[37]^ In this study, the overall pairwise comparisons between the groups did not identify statistically significant differences regarding the MAP and HR except the period laryngeal mask extraction. In regards to the heart rate, after induction into anesthesia, it was significantly lower. Then, with the establishment of the pneumoperitoneum, the heart rate faster and there was no statistically significant difference between time endpoints explaining the effect from the low intra-abdominal pressure and the mild reverse Trendelenburg position which probably improved the venous return. When patients adapted to ketorolac tromethamine and dezocine, the HR and MAP remained stable.

### 4.1 Limitations

First, this study is a single-center study, and more cases in multiple centers are needed to verify the conclusions of this study; Second, in this study, only male patients undergoing laparoscopic inguinal hernia repair were selected, and the effects of other types of surgery and female patients were not verified; Third, the postoperative follow-up time of this study is short, only up to 24 hours after the operation, and longer follow-up is needed to determine the final effect.

## 5 Conclusion

Ketorolac tromethamine combined with dezocine can to a certain extent reduce the use of conventional anesthesia drugs during general anesthesia-assisted inguinal hernia repair, reduce the cardiovascular reaction during extubation during anesthesia recovery, and benefit patients’ postoperative sedation and analgesia.

## Author contributions

**Conceptualization:** Jinbao Wang, Yu Wu.

**Data curation:** Bohan Fu, Jinbao Wang.

**Formal analysis:** Bohan Fu, Yanli Li, Yuling Kang, Jinbao Wang.

**Funding acquisition:** Yu Wu.

**Investigation:** Bohan Fu, Zenghua Cai, Jinbao Wang.

**Methodology:** Zenghua Cai.

**Resources:** Yanli Li, Yuling Kang.

**Software:** Yanli Li, Yuling Kang.

**Supervision:** Yu Wu.

**Writing – original draft:** Jinbao Wang, Yuling Kang.

**Writing – review & editing:** Yu Wu.
